# Choline Alphoscerate: A Therapeutic Option for the Management of Subthreshold Depression in the Older Population

**DOI:** 10.3390/geriatrics10020032

**Published:** 2025-02-20

**Authors:** Nicolò Granata, Marco Vercesi, Annamaria Bonfanti, Claudio Mencacci, Ilaria Coco, Mario Mangrella, Roberto Piazza, Giancarlo Cerveri

**Affiliations:** 1 Dipartimento di Salute Mentale e Dipendenze, ASST Lodi, 26900 Lodi, Italy; nicolo.granata@asst-lodi.it (N.G.); marco.vercesi@asst-lodi.it (M.V.); giancarlo.cerveri@asst-lodi.it (G.C.); 2 Dipartimento di Scienze Mentali e Neuroscienze, ASST Fatebenefratelli, 20157 Milano, Italy; claudio.mencacci@gmail.com; 3 Medical Affairs Department, Italfarmaco S.p.a., 20092 Milan, Italy; i.coco@italfarmacogroup.com (I.C.); m.mangrella@italfarmacogroup.com (M.M.); r.piazza@italfarmacogroup.com (R.P.)

**Keywords:** subthreshold depression, older, major depressive disorder, choline alphoscerate, L-alpha-glycerylphosphorylcholine

## Abstract

**Background and Objectives:** Subthreshold depression (StD) presents with depressive symptoms similar to major depressive disorder (MDD) but of lower intensity. Despite its milder form, StD is significantly prevalent in the older population, affecting up to 12.9%. StD is associated with adverse outcomes, such as an increased risk of MDD and mild cognitive impairment (MCI). Treating StD in older adults is challenging due to the limited efficacy and side effects of traditional antidepressants. As a result, clinicians often adopt a “watchful waiting” strategy, which increases the risk of StD progressing into MDD or MCI. Choline alphoscerate (α-GPC), a cholinergic drug, is indicated in the treatment of pseudodepression in the elderly, a condition that corresponds to the actual definition of StD. This review highlights the role of α-GPC in the treatment of StD in older subjects. **Methods:** A comprehensive review of preclinical and clinical studies was conducted, focusing on the efficacy of α-GPC in improving cognitive and behavioral functions in mental conditions and in modulating neurotransmitter systems involved in depression, such as dopamine and serotonin. **Results:** Evidence points to the therapeutic benefits of using α-GPC in StD as it acts on cholinergic dysfunction and cognitive impairment. Additionally, it may improve mood regulation and motivation, key factors in StD and in depressive disorders. These findings suggest that α-GPC may reduce the risk of progression from StD to MDD or MCI. **Conclusions:** α-GPC represents an effective and safe therapeutic option for the treatment of StD in the older population, improving clinical outcomes and enhancing the quality of life in this high-risk group.

## 1. Introduction

Subthreshold depression (StD) is characterized by clinically significant depressive symptoms (CSDSs) that do not meet the criteria for major depressive disorder (MDD) [1,2] or dysthymia [3,4]. 

Despite its less severe presentation, StD imposes a greater burden on health services than MDD [5] because of its higher prevalence, estimated at 11.02% [6]. 

StD can manifest with the same symptoms found in MDD, including a depressed mood, diminished interest or pleasure in activities, feelings of worthlessness or excessive guilt, impaired concentration, fatigue, psychomotor changes, sleep disturbances, altered appetite, and suicidal ideation [7]. 

However, the differences lie in the number, duration, severity, and prevalence of these symptoms, as well as the specific exclusion criteria [7]. Although less severe than MDD-associated manifestations, these symptoms are nonetheless distressing and can severely impact quality of life [8].

In the older population, StD is particularly prevalent, with rates reaching 12.9% [6], at least 2–3 times higher than those of MDD [9]. Moreover, depressive symptoms in older adults often present atypically, with sadness, apathy, non-specific pain, fatigue, dyspnea, dizziness, and cognitive complaints, such as memory loss, which are frequently overlooked or misdiagnosed [10,11,12].

StD has garnered increasing attention from the scientific community because of its high prevalence and association with adverse outcomes in later life [6,9,13,14], including a heightened risk of MDD [6,8] and mild cognitive impairment (MCI) [15]. However, clear guidelines for the clinical management of StD are lacking, and there is considerable debate regarding the effectiveness of various treatment strategies. These strategies range from watchful waiting to psychotherapy, herbal remedies such as St. John’s wort, and psychopharmacological therapies with antidepressants [16]. Currently, older patients with StD are often advised to undergo watchful waiting, which delays effective treatment and increases the risk of progression to MDD or cognitive impairment. Alternatively, they may be prescribed antidepressants, whose efficacy in treating StD remains uncertain [17,18], as many patients fail to respond to treatment [17]. Furthermore, the potential side effects, such as an increased risk of dementia, accidents, and falls, are particularly concerning in the older population [19,20,21,22,23,24,25,26,27,28]. For these reasons, there is a pressing need to develop and evaluate primary prevention strategies that reduce the risk of StD evolving into a chronic condition with a high risk of recurrence [29], especially in older adults who represent a high-risk group [30].

Choline alphoscerate, or L-alpha-glycerylphosphorylcholine (α-GPC), is a cholinergic drug with well-established efficacy and safety profiles approved for the treatment of pseudodepression in elderly individuals, a condition that corresponds to the actual definition StD. Known for its neuroprotective properties, α-GPC supports cognitive function and has been shown to improve memory and cognitive recovery in various neurodegenerative and vascular conditions, including MCI, dementia, Alzheimer’s disease (AD), acute stroke, and transient ischemic attacks [31,32,33,34,35]. Knowing that the cognitive symptoms of depression, such as impaired attention, concentration, and memory, are linked to cholinergic dysfunction [36,37], it is plausible that α-GPC can address the cholinergic deficits associated with depression [36].

This review aims to consolidate evidence from preclinical and clinical studies regarding the efficacy of α-GPC in treating cognitive impairment in neurological conditions, such as AD, Parkinson’s disease (PD), and vascular dementia (VaD). Additionally, it examines the role of α-GPC in addressing the widespread occurrence of StD in older individuals [9,38,39,40,41,42], highlighting its value as a tool to prevent the onset of MDD or cognitive decline, for which StD is a known risk factor [43,44,45]. This approach presents a pathway to enhance both the clinical outcomes and quality of life in this population.

## 2. Methods

We conducted an online search for articles on StD. Relevant articles were retrieved from PubMed using the search terms “subthreshold depression”, “prevalence”, “treatment”, and “older adults” in several combinations. Articles were initially screened by title and abstract for relevance, followed by a full-text review. Additionally, the reference lists of selected articles were scanned to identify further pertinent studies in the literature. Original articles in English were included from inception to May 2024.

## 3. Subthreshold Depression in the Older Population

The definitions of StD vary significantly, reflecting its complex nature. According to the *Diagnostic and Statistical Manual of Mental Disorders, Fifth Edition* [46], StD can be viewed as a distinct disorder, a continuum with MDD [47,48], or a state characterized by residual depressive symptoms indicative of incomplete remission [49]. Recently, Volz et al. proposed defining StD as the presence of at least two DSM depressive symptoms for at least two weeks; one symptom of depressed mood without meeting the criteria for MDD or minor depression; a Brief Patient Health Questionnaire Mood Scale (PHQ-9) score between 5 and 9 and/or a Centre for Epidemiological Studies Depression Scale-Revised (CESD) score of at least 16; and Montgomery–Ӑsberg Depression Rating Scale (MDRS) score between 10 and 18 (for 2 weeks) [50]. 

StD corresponds to “pseudodepression” in the elderly considering that the clinical manifestations are the same. StD may act as both a precursor to and a consequence of MDD [7], with conversion rates from StD to MDD in older adults estimated between 20 and 30% [43,44,45]. However, precisely determining how many individuals with StD will progress to MDD is difficult because of the variability in StD definitions [8]. For instance, a 6-year longitudinal study conducted by Cuijpers et al. [44] revealed that 7.8% of older adults with StD developed MDD, while another study in the Netherlands [51] reported a rate of 12% over 3 years.

Nearly 14% of individuals over the age of 55 experience a depressive syndrome, but only 2% meet the criteria for MDD [52]. StD is more prevalent than both MDD and dysthymia, affecting approximately 10% of community-dwelling older adults (compared with 1–4% for MDD), 20% of primary care patients (compared with 5–10% for MDD), and up to 30% of hospitalized patients and nursing home residents (compared with 10–12% for MDD) [9,38,39,40,41]. The incidence of StD ranges from 2.9% to 9.9% among adults in primary care, 1.4% to 17.2% in community settings [53], and as high as 38.7% among older adults receiving home care [30]. Furthermore, 8–16% of older adults exhibit CSDSs, a term often used interchangeably with StD [40]. In contrast, dysthymia affects around 2% of the older population [3]. The Pietà study revealed that 26.5% of community-dwelling individuals aged 75 years and older have experienced CSDSs [42].

The risk factors for StD closely mirror those for depressive disorders, including the female sex, childhood poverty, a low socioeconomic status, a lack of social support, abuse, personal loss or negative life events, personal or family history of depression or other psychiatric disorders, physical disability, loneliness, insomnia, and clinical comorbidities [7].

StD poses a significant health burden for older adults [54], being more prevalent than clinically diagnosed depression [55]. It adversely impacts social functioning [56], activities of daily living [57], and overall quality of life [58]. In addition to increasing the risk of developing MDD [6], StD is associated with a heightened risk of cognitive impairment and dementia: nearly 20% of patients with MCI suffer from StD [15]. 

StD is also linked to a worsening of physical ailments [59], functional decline [59], increased mortality [60], and higher medical costs [6,9,14,61]. In older adults, StD exacerbates the burden of chronic diseases such as angina, arthritis, asthma, and diabetes, increasing both morbidity and mortality [62,63]. This condition contributes to the transition of older adults from community living to primary care settings and eventually to long-term care facilities [9].

Despite its prevalence and impact, StD is often underdiagnosed in older adults due to underreporting by patients and the presence of confounding comorbidities [7,64].

## 4. Clinical Management of Subthreshold Depression

Managing StD requires a comprehensive approach. Diagnosis should involve a thorough patient history, including an evaluation of previous depressive episodes, qualitative and quantitative assessments of current symptoms, the use of categorical scales and classifications, and a review of past and present antidepressant use [7]. Regular reassessments are essential, particularly to identify whether StD is a prodromal phase of MDD [7].

Various intervention strategies can be considered, including watchful waiting, psychotherapy, herbal remedies such as St. John’s wort, and pharmacological therapies with antidepressants [16]. However, there is ongoing debate about the effectiveness of these approaches [16].

### 4.1. Non-Pharmacological Interventions

Electroacupuncture has gained attention as a treatment for psychiatric disorders, including depression [65,66,67]. It modulates the hypothalamic–pituitary–adrenal axis [68] and restores hippocampal CA1 synaptic plasticity by adjusting serotonin receptor levels, thus exhibiting antidepressant effects and improving depression-like symptoms [69] while maintaining a favorable safety profile [70].

Watchful waiting involves monitoring the patient without active treatment, focusing on the assessment of symptoms, their progression, and the overall impact [71]. This approach may be suitable for individuals with strong social support, no history of depressive disorders, and those who decline medication or psychotherapy after understanding the risks and treatment options [71]. However, watchful waiting alone may be insufficient given the risk of progression from minor to major depression, functional impairment, and diminished quality of life, even in StD [16,72,73].

Herbal remedies, particularly St. John’s wort, are popular among patients who prefer natural treatments over conventional medications. However, its efficacy remains controversial, with many studies showing no significant benefits over placebo in treating major or minor depression [74,75,76].

Psychotherapy is increasingly being recognized as a critical treatment option for StD [77], with strong evidence supporting its use as a first-line intervention [78,79]. While higher-intensity therapies, such as cognitive–behavioral therapy (CBT), interpersonal therapy, and behavioral activation, are typically reserved for more severe disorders [80], recent systematic reviews have shown the effectiveness of CBT and other forms of therapy in alleviating StD symptoms compared with control groups [77,81]. However, barriers to accessing traditional psychotherapy include time, cost, stigma, and limited provider availability [82]. These issues have been exacerbated by the increased demand for mental health services due to the impact of the COVID-19 pandemic, especially in low- and middle-income countries [83].

Interestingly, psychotherapy has shown high efficacy in treating comorbid conditions such as depression and poor glycemic control, underscoring its versatility [84].

Non-pharmacological interventions with proven efficacy in MDD may also benefit individuals with StD. Exercise interventions, including dance movement therapy, have been shown to significantly reduce depressive symptoms compared to usual care in individuals with depression [85,86,87]. The most pronounced benefits were observed in high-intensity exercise programs lasting 12 weeks or less, particularly when sessions were supervised [85]. These findings underscore the potential of structured, short-term, high-intensity exercise as an effective strategy for managing depressive symptoms in individuals with StD. 

Higher levels of mindfulness may alleviate symptoms of depression and anxiety by enhancing emotional regulation strategies, particularly by reducing maladaptive processes, such as worry and rumination [88].

Patient preferences play a critical role in the management of StD. Educating patients about their conditions and available treatment options facilitates shared decision-making between physicians and patients. Research has demonstrated that respecting patient preferences leads to better adherence, higher treatment satisfaction, and improved outcomes [7].

### 4.2. Pharmacotherapy: Antidepressants 

The prevailing hypothesis for depression’s pathogenic mechanism focuses on the role of monoamine neurotransmitters [89]. Pharmacotherapy typically includes selective serotonin reuptake inhibitors, noradrenaline reuptake inhibitors, serotonin and noradrenaline reuptake inhibitors, tricyclic antidepressants (TCAs), and monoamine oxidase inhibitors [90,91]. However, these medications generally require 2–4 weeks to show effects [92], and approximately 40% of patients with depression do not respond to these treatments [93,94].

In older adults, there is growing evidence that antidepressant use is associated with significant side effects, including weight gain [95], cognitive impairment [27,96,97], falls [25,98,99,100], syndrome of inappropriate antidiuretic hormone secretion [101], and serotonin syndrome [102]. TCAs are generally considered inappropriate for older patients, regardless of pathology, because of their strong anticholinergic effects, their potential to cause cognitive impairment and worsening comorbidities, and cardiotoxic risks [27]. Furthermore, the use of benzodiazepines in older adults is associated with increased risks of dementia [20], traffic accidents [19], confusion [21,22], falls [21,22], and, in rare cases, a paradoxical increase in agitation [23]. These symptoms have prompted experts to exercise caution when prescribing traditional antidepressants to this vulnerable population [103]. 

Antidepressants are not recommended as a first-line treatment for StD due to limited evidence of their efficacy. A systematic review found no significant advantage of antidepressants over placebo in patients with minor depression [104]. Similar findings have emerged from randomized trials [16], while others have supported the superiority of antidepressants over placebo [16,105]. While some studies suggest that antidepressants may be more beneficial for severe depression, their effectiveness in treating StD remains uncertain [17,18]. The side effects and unclear efficacy make these treatments less desirable for StD.

## 5. Choline Alphoscerate: A Therapeutic Option for Subthreshold Depression

The delayed and, in many cases, poor response to current antidepressants suggests that additional neurotransmitters may be involved in the pathophysiology of depression beyond those accounted for by the monoamine theory. 

Disrupted glutamatergic neurotransmission and impaired regulation of the glutamine–glutamate cycle may contribute to the pathophysiology of depression in elderly individuals [106]. Optimizing neuromodulatory interventions, such as transcranial magnetic stimulation and transcranial direct current stimulation, to specifically target glutamatergic pathways may enhance their efficacy in treating depression [107]. 

Notably, cholinergic and glutamatergic pathways interact, and D-serine has been suggested to play a role in this connection [108]. Stimulation of the nucleus basalis of Meynert, responsible for cortical cholinergic innervation, significantly increases D-serine levels [109], and D-serine modulates NMDA receptor activity [110,111]. Additionally, choline alphoscerate enhances cholinergic neurotransmission [112], potentially reducing glutamate-induced neuronal damage by stimulating nicotinic acetylcholine receptors and activating the phosphatidylinositol 3-kinase signaling pathway [113].

The cholinergic hypothesis of geriatric memory dysfunction suggests that a loss of cholinergic function in the central nervous system plays a significant role in the cognitive decline associated with aging and AD [114]. This manifests in symptoms, such as poor attention, concentration, impaired memory, and slowed information processing, largely due to disruptions in adult neurogenesis and hippocampal function, an area rich in cholinergic innervations [36,115,116]. These disruptions may also contribute to the development of apathy [117]. Many drugs used to treat comorbidities in older patients have anticholinergic properties, which can further exacerbate cholinergic dysfunction [118,119]. As a result, symptoms stemming from reduced cholinergic neurotransmission, either directly or indirectly, could potentially be alleviated by strategies to enhance cholinergic transmission and increase acetylcholine bioavailability [31,32,33,34,35,120,121,122,123,124,125,126]. Several approaches have been explored to address cholinergic deficiencies in cognitive decline [31,32,33,34,35,120,121,122,123,124,125,126]. Clinical studies using muscarinic acetylcholine receptor agonists, acetylcholinesterase inhibitors (AChEIs), and acetylcholine-releasing agents have demonstrated some efficacy in treating patients with AD or dementia [127,128,129,130,131,132]. However, while AChEIs can provide limited cognitive improvements in patients with AD, they do not halt the disease’s progression [128,129,133]. Nonetheless, AChEIs remain the first line of pharmacological treatment for cognitive deficits in dementia and AD [127,128,129,130].

An experimental observation revealed that under conditions of reduced cholinergic synthesis and increased neuronal demand, neurons increase their capacity to incorporate exogenous choline [134]. This suggests that administering a choline precursor can counteract cholinergic deficiencies and thus improve cognitive function.

Among cholinergic precursors, cytidine 5′-diphosphocholine (CDP-choline) and α-GPC have demonstrated the ability to enhance acetylcholine content and release [135,136,137]. These agents cross the blood–brain barrier efficiently [138] in contrast to first-generation acetylcholine precursors, such as choline and phosphatidylcholine (lecithin), which were shown to be ineffective in dementia treatment [125].

Among these, α-GPC is considered the most effective source of choline, containing 41% choline by weight [138], and outperforms CDP-choline in elevating plasma choline levels, as well as in acetylcholine synthesis and release [135,137]. 

Once administered orally, α-GPC is metabolized into phosphatidylcholine, the active form of choline [31,139]. It raises free plasma choline more rapidly than other uncharged precursors and becomes incorporated into brain phospholipids within 24 h [140]. This process allows α-GPC to enhance acetylcholine levels and support the cholinergic structures in the brain, particularly those in the basal forebrain that are sensitive to ischemic damage [125] and in cases of choline deficiency [31,139]. Studies confirmed that α-GPC reaches the brain whether administered orally or by injection [141]. Acetylcholine is vital for neural communication, playing a key role in storing and recalling information [142]. Additionally, α-GPC promotes the release of brain-derived neurotrophic factor, which is essential for neuroplasticity and cognitive health [143,144]. 

### 5.1. Preclinical Studies on α-GPC

Preclinical research has shown that α-GPC enhances acetylcholine release in the hippocampi of rats [135,145,146], facilitates learning and memory processes, counteracts cognitive deficits in experimental models of aging, and reverses memory impairments induced by scopolamine administration [35,135,146,147]. Furthermore, α-GPC mitigates cognitive impairment and cell damage in rodent models subjected to hippocampal irradiation [148] and prevents age-related changes in brain microanatomy and impairments of cholinergic neurotransmission markers and receptors occurring in rats [147]. α-GPC treatment reduced cortical and hippocampal reactive astrocytes and pro-inflammatory microglia, increasing anti-inflammatory activity, with a positive effect on the synaptic marker synaptophysin in the hippocampus [149]. One notable study on adult male rats revealed that combining α-GPC with the cholinesterase inhibitor rivastigmine significantly increased both acetylcholine levels and [3H]hemicholinium-3 binding [112]. This effect was more pronounced in the hippocampus than in the prefrontal cortex and helped prevent volume loss in critical brain areas, such as the frontal and temporal lobes, hippocampus, and striatum [112].

Additionally, α-GPC has been found to stimulate hippocampal neurogenesis and provide neuroprotective effects, shielding against neuronal death and cognitive decline in animal models of pilocarpine-induced seizures [150].

In studies on spontaneously hypertensive rats, Tayebati and colleagues [151] found that combining α-GPC with AChEI galantamine produced greater neuroprotective effects than administering either compound alone. In addition, α-GPC treatment prevented hypertension-related neuronal loss in the hippocampus, a key area for learning and memory, along with the accompanying glial reaction [152]. 

The increase in cholinergic transmission facilitated by α-GPC may help prevent glutamate neurotoxicity via the activation of nicotinic acetylcholine receptors and the phosphatidylinositol 3-kinase pathway [113,153,154].

Additional benefits of α-GPC include its ability to stimulate the dopaminergic system [155], which plays a well-documented role in the regulation of apathy [156]. It has also been shown to increase serotonin levels in the frontal cortex and striatum of rat brains [155], suggesting that α-GPC may influence multiple neurotransmitter systems.

### 5.2. Clinical Studies on α-GPC

α-GPC has shown to be effective in improving memory and promoting cognitive recovery in several neurodegenerative and vascular diseases, including dementia, AD, acute stroke, post-COVID-19 or post-traumatic cognitive impairment, and transient ischemic attacks (Table 1) [31,32,33,34,35,120,121,122,123,124,125,126]. Specifically, the benefits associated with α-GPC in patients with dementia include improved orientation, attention, memory, language, and mood [120]. An increase in acetylcholine levels in the aging brain has been found [120] to have a notable impact on memory and attention deficits [125]. It has also been observed that α-GPC prevents ischemia-induced oxidative stress and inflammation by preserving mitochondrial respiratory function [120]. 

In a clinical trial involving 261 patients with mild to moderate AD-related dementia, treatment with α-GPC significantly slowed cognitive decline and improved symptoms, whereas the placebo group experienced no change or worsening of symptoms [32]. A systematic review also reported that between 9.4% and 38.8% of patients with AD exhibit minor depressive disorders, including StD [157].

A multicenter randomized controlled study compared α-GPC with acetyl-L-carnitine (ST-200) in 126 patients with probable Alzheimer-type senile dementia (mild to moderate) [124]. Both treatments resulted in comparable improvement in the Mini-Mental State Examination (MMSE) scores [124]. In the αGPC group, significant improvements were recorded in verbal memory, intellectual and emotional impairments, confusion, and depression [124]. The Gottfries–Bråne–Steen (GBS) Rating Scale [158] showed statistically better outcomes in the α-GPC group than in the ST-200 group after 6 months [124]. In addition, Sandoz Clinical Assessment Geriatrics (SCAG) factors, including “affective disorders”, “apathy”, “somatic functioning”, and “overall impression”, showed significant improvements in the α-GPC group compared with the ST-200-treated group [111]. Adverse effects in the α-GPC-treated group included insomnia, gastralgia, and restlessness, each of which occurred in one patient [124].

While treatments such as AChEIs (donepezil, galantamine) and the N-methyl-D-aspartate receptor antagonist memantine have shown modest cognitive improvements in patients with VaD, their functional and global benefits remain inconsistent, with evidence from only two large trials on donepezil [159]. 

α-GPC has demonstrated positive effects on cognitive performance and a good tolerability profile in patients with vascular cognitive impairment [122,160,161,162]. One study involving 789 patients with VaD showed that α-GPC improved memory, attention, and affective and somatic symptoms (such as fatigue and vertigo), with outcomes being superior to the placebo and comparable to or better than reference compounds, such as CDP-choline [120,125].

The CONIVaD (Choline Alphoscerate and Nimodipine in Vascular Dementia) study aimed to evaluate the efficacy and safety of combining α-GPC with nimodipine in patients with cerebral small vessel disease (SVD) and mild to moderate cognitive impairment [163]. Although treatment adherence was low, the combination did not show significant effects, but the safety profile was deemed good overall [163].

Further studies have highlighted the positive role of α-GPC in recovering cognitive function after cerebrovascular accidents [164,165]. An Italian multicenter trial involving 2044 patients recovering from stroke or transient ischemic attacks showed that α-GPC contributed to cognitive recovery and was well tolerated [31].

Cognitive impairments are also among the most common non-motor symptoms of PD. AChEIs, particularly rivastigmine, have shown efficacy in controlled trials for patients with PD and dementia, as has memantine, although their effectiveness is considered moderate [166,167]. In a separate open-label study [168], α-GPC demonstrated superior efficacy to piracetam in improving cognitive function in patients with PD. Marked and moderate cognitive improvements were observed with α-GPC in 40% of patients compared with piracetam in 25% of patients [157]. The incidence of cognitive deterioration was lower in patients treated with α-GPC (5%) compared with piracetam (15%, *p* < 0.05) [168]. Additionally, α-GPC treatment led to a decrease in behavioral disturbances, particularly apathy [168]. Overall, α-GPC was very well tolerated by the patients [168].

In younger populations, α-GPC reversed scopolamine-induced amnesia in healthy volunteers [169]. A recent randomized, double-blind, placebo-controlled trial reported a decrease in the Alzheimer’s Disease Assessment Scale–Cognitive Subscale (ADAS-cog score) in overall healthy subjects aged 55–85 years old with amnestic mild cognitive impairment treated with α-GPC [170]. A study on the supplemental effects of α-GPC in hearing aid users found that the reduced activation of nicotinic acetylcholine receptors in the medial geniculate body of the aging brain contributes to declining speech detection, recognition, and comprehension [171]. In such cases, supplementation with choline precursors may help improve language function [171].

The efficacy of α-GPC was also explored in combination with AChEI to potentiate its cholinergic activity (Table 2). A systematic review and meta-analysis found that α-GPC, either alone or combined with donepezil, improved cognitive, behavioral, and functional outcomes in patients with neurological conditions associated with cerebrovascular injury [123]. α-GPC also showed greater efficacy in combination with AChEIs [126,151], as demonstrated in the ASCOMALVA trial [156,172,173,174,175] involving 113 patients with mild to moderate AD. Patients receiving both donepezil and α-GPC exhibited fewer behavioral disturbances, reduced caregiver stress, and lower scores for depression and apathy than those treated with donepezil alone [156,172,173,174,175]. This combination therapy significantly reduced behavioral and psychological symptoms of dementia and alleviated caregiver distress, particularly by improving mood disorders such as depression and anxiety [172] in patients with mild to moderate cognitive impairment [173].

Combining α-GPC with AChEIs has also been shown to significantly increase brain acetylcholine concentrations and prevent volume loss in the frontal and temporal lobes, hippocampus, and amygdala of patients with AD [176].

The advantage of this combination is that lower doses of AChEIs are required to increase brain acetylcholine levels, reducing the risk of gastrointestinal and hepatic side effects typically associated with AChEI monotherapy [177].

Overall, most studies report positive effects of α-GPC on cognitive function and emphasize its efficacy in cognitive enhancement, particularly when used in combination with other therapeutic agents [178], with excellent tolerance and minimal side effects, typically mild in nature, at doses of up to 1200 mg/day [32,120,125,170]. However, one large-scale study raised concerns about an increased risk of stroke, highlighting the need for further investigations into the safety profile of α-GPC, particularly in older individuals with multiple comorbidities [179]. Interestingly, a recent systematic review and meta-analysis reported improved neurological function and functional recovery in patients who experienced stroke [180]. A randomized controlled trial involving 60 participants is currently underway to confirm the efficacy and safety of α-GPC in slowing or stabilizing brain atrophy and in improving or slowing the progression of cognitive and behavioral manifestations in patients aged 65 years and older with mild cognitive dysfunction associated with vascular damage [181].

**Table 1:** Summary of studies evaluating choline alphoscerate for cognitive and behavioral outcomes.

Study	Study Design	Disease	N	Treatment	Efficacy Measures	Advantages of α-GPC Treatment	Disadvantages of α-GPC Treatment/Study Limitations
Di Perri et al. [122] (1991)	RCT	Multi-infarct dementia	120	α-GPC (1000 mg/day, parenteral, 90 days) vs. CDP-choline	Modified PBRS, SCAG, HRSD	Comparable or improved efficacy vs. CDP-choline (varies by clinical domain); good/optimal tolerability reported by 93.3% of patients	Limited follow-up
Canal et al. [169] (1991)	RCT	Scopolamine- induced amnesia	32	α-GPC (400 mg/day, oral pretreatment, 10 days vs. placebo) before scopolamine	Memory tests	Rapid onset of memory improvement	Small sample; artificial experimental condition
Parnetti et al. [124] (1993)	RCT	AD-type senile dementia	126	α-GPC (1200 mg/day, oral, 180 days) vs. ST-200	GDS, SCAG, clinical symptoms, GBS, MMSE, Rey’s 15-words test	Improved GBS, GDS, and SCAG scores compared to ST-200; mild AEs in 4.6% of patients	Limited to AD dementia cases
Barbagallo Sangiorgi et al. [31] (1994)	Prospective cohort study	Cerebral ischemic attacks	2044	α-GPC (1000 mg/day, parenteral, 28 days, and then 1200 mg/day, oral, 6 months)	Mathew/MMSE, CRS, GDS, clinical recovery	Improved cognitive recovery after ischemic attack; mild AEs in 2.14% of patients	Short study duration; limited to Italian centers
Parnetti et al. [120] (2001)	Analysis of published data	Cognitive decline	1570 (10 trials)	α-GPC (1200 mg/day, oral, 3–6 months or 1000 mg/day, parenteral, 3 months)	Various tests assessing mental deterioration, including MMSE	Improved clinical condition; memory and attention impairment	2 uncontrolled trials (854 patients) out of 10
		Acute cerebrovascular dementia	2484 (3 trials *)	α-GPC (1000 mg/day, parenteral, 4 weeks, and then 1200 mg/day, oral, 5 months)	Mathew/MMSE, GDS, CGRS	Improved cognitive, functional, and motor outcomes	Uncontrolled trials
De Jesus Moreno [32] (2003)	RCT	Mild to moderate AD dementia	261	α-GPC (1200 mg/day, oral, 180 days) vs. placebo	ADAS-Cog, MMSE, GDS, ADAS-Behav, ADAS-Total, CGI, GIS	Significant cognitive improvement vs. placebo; mild AEs in 8.3% of patients	Limited to mild to moderate cases; small sample size
Levin et al. [168] (2011)	Prospective cohort study	PD with cognitive impairment	60	α-GPC (Cereton, 1200 mg/day, oral, 12 weeks) vs. piracetam	CGI, MMSE, motor/cognitive outcomes	Improved psychological status, attention, and regulatory cognitive functions; mild and short-term AEs in 15% of patients	Small sample size; PD-specific focus
Na et al. [171] (2021)	Prospective cohort study	Age-related hearing loss	34	α-GPC (800 mg/day, oral, 11 months)	Speech recognition tests	Improved speech recognition in older adults	Small sample size; limited to hearing impairment
Lee et al. [179] (2021)	Retrospective cohort study	Stroke risk association (long-term)	108,877 α-GPC users	α-GPC	Stroke incidence		Association of α-GPC with higher risk of stroke; α-GPC users older and with more comorbidities; potential underestimation of stroke events
Sagaro and Amenta [180] (2023)	Systematic review *	Acute and hemorrhagic stroke	8357 (15 studies); 5 studies include α-GPC users	α-GPC (1000 mg/day, parenteral—1200 mg/day, oral) vs. citicoline	Mathew/MMSE	Choline alphoscerate improved neurological function and functional recovery and reduced dependency in patients who experienced stroke	Uncontrolled trials; limited number of RCTs

* The analysis includes the paper by Barbagallo Sangiorgi et al. [31] (1994). ADAS-Behav: Alzheimer’s Disease Assessment Scale–Behavioral Subscale; ADAS-Cog: Alzheimer’s Disease Assessment Scale–Cognitive Subscale; ADAS-Total: all items of the Alzheimer’s Disease Assessment Scale; AD: Alzheimer’s disease; AEs: adverse events; α-GPC: choline alphoscerate; CGI: Clinical Global Impression; CGRS: Crichton Geriatric Rating Scale; CRS: Crichton Rating Scale; GBS: Gottfries–Brane–Steen Rating Scale for Dementia; GDS: Global Deterioration Scale; GIS: Global Improvement Scale; HRSD: Hamilton’s Rating Scale of Depression; MMSE: Mini-Mental State Test; PBRS: Parkside Behaviour Rating Scale; PD: Parkinson’s disease; RCT: randomized controlled trial; SCAG: Sandoz Clinical Assessment Geriatric Scale.

**Table 2:** Summary of studies evaluating choline alphoscerate in combination therapy for cognitive and behavioral outcomes.

Study	Study Design	Disease	N	Treatment	Efficacy Measures	Advantages of α-GPC Treatment	Disadvantages of α-GPC Treatment /Study Limitations
Amenta et al. [175] (2012)—ASCOMALVA	RCT (interim results)	AD with cerebrovascular injury	91	α-GPC (1200 mg/day, oral, 1 year) + donepezil vs. donepezil alone	MMSE, NPI, ADAS-Cog, BADL, IADL	Improvement in analyzed items (except BALD) compared to donepezil alone; combination treatment tolerability comparable to donepezil group	Limited to interim results; no final outcome data
Amenta et al. [174] (2014)—ASCOMALVA	RCT (interim results)	AD with cerebrovascular injury	113	α-GPC (1200 mg/day, oral, 2 years) + donepezil vs. donepezil alone	MMSE, NPI, ADAS-Cog, BADL, IADL	Improved cognitive test performance and BADL, IADL, and NPI scores (including caregiver distress) compared to donepezil alone; tolerability of combination treatment comparable to donepezil group	Study still ongoing at time of publication
Rea et al. [156] (2015)—ASCOMALVA trial	RCT (interim results)	AD with apathy	113	α-GPC (1200 mg/day, oral, 2 years) + donepezil vs. donepezil alone	Apathy subtest of NPI	Lower apathy score and caregiver distress compared to donepezil alone	Retrospective collection of data; limited sample size; measurement of apathy with one scale (NPI)
Carotenuto et al. [172] (2017)—ASCOMALVA	RCT (interim results)	AD with BPSD	113	α-GPC (1200 mg/day, oral, 2 years) + donepezil vs. donepezil alone	MMSE, NPI, ADAS-Cog, BADL, IADL	Lower levels of behavioral disturbances compared to donepezil alone	Retrospective collection of data; limited sample size
Traini et al. [176] (2020)—ASCOMALVA	RCT (interim results)	AD	56	α-GPC (1200 mg/day, oral, 3 years) + donepezil vs. donepezil alone	MRI volume analysis of cognitive areas	Improved cognitive function, BADL, and NPI scores; reduced gray matter loss and brain atrophy; significantly lower hippocampal volume reduction; tolerability comparable to donepezil group	Small sample size; limited to interim results
Carotenuto et al. [173] (2022)—ASCOMALVA	RCT	AD with depression	90	α-GPC (1200 mg/day, oral, 3 years) + donepezil vs. donepezil alone	MMSE, ADAS-Cog, NPI	Reduced depression symptoms compared to donepezil alone	Retrospective collection of data; limited sample size; measurement of depression with one scale (NPI)
Kang et al. [126] (2022)	Retrospective cohort study	Mild to moderate dementia (>70% AD dementia)	583 (74 α-GPC)	α-GPC (12 months) + AChEI	MMSE	Improved MMSE language subscale scores	Lack of randomization (real-world data)

AchEI: acetylcholinesterase inhibitors; AD: Alzheimer’s disease; ADAS-Cog: Alzheimer’s Disease Assessment Scale–Cognitive Subscale; BADL: basic activities of daily living; BPSD: behavioral and psychological symptoms of dementia; IADL: instrumental activities of daily living; MMSE: Mini-Mental State Test; NPI: Neuropsychiatric Inventory; RCT: randomized controlled trial.

## 6. Experts’ Perspective

A growing consensus among experts suggests that using acetylcholine precursors, such as α-GPC, can offer significant benefits in StD. α-GPC is better tolerated than AChEIs and has shown greater efficacy in clinical studies for improving both cognitive and behavioral symptoms, especially compared with other precursors such as CDP-choline [161]. The ability of α-GPC to effectively elevate plasma choline levels and boost acetylcholine synthesis and release may explain these superior outcomes [135].

α-GPC represents a therapeutic opportunity to improve cognitive function while maintaining a favorable safety profile [32,120,125]. By increasing cholinergic activity early on, α-GPC can help address the underlying dysfunction contributing to cognitive decline and behavioral symptoms, such as apathy [155,172,173,174,175].

Besides its role in memory enhancement and cognitive recovery in several neurological diseases [31,32,33,34,35,120,121,122,123,124,125,126], α-GPC shows potential in modulating other neurotransmitter systems, particularly dopamine and serotonin [155]. Dopamine transmission is strongly associated with motivation and reward processing [182,183,184], while the serotonergic system is mainly related to anxiety and depression [185,186]. This highlights its broader applicability, especially in treating StD, a condition often found in older adults but frequently undertreated. 

Unlike antidepressants, which are associated with uncertain efficacy and the aforementioned side effects, α-GPC offers a safer and well-tolerated alternative, particularly in older patients with StD symptoms including apathy. This condition primarily affects volition and may coexist with mood disorders, such as depression [187], and with early cognitive decline. The symptomatology of pseudodepression and StD in the older population overlap.

**Figure 1:**
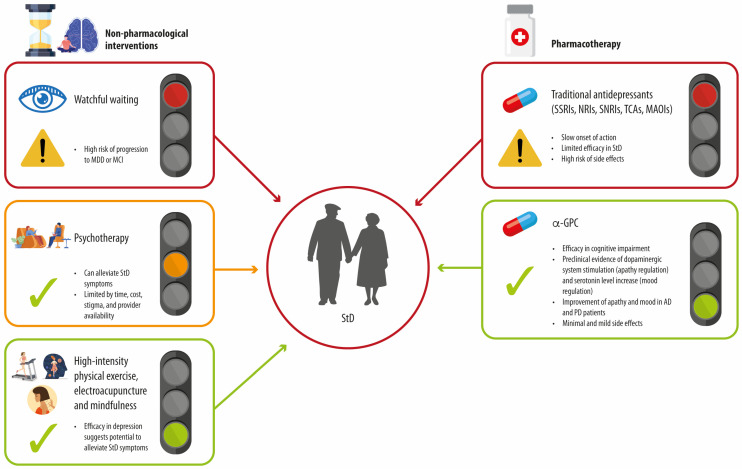
Summary of treatment strategies commonly employed by clinicians for managing StD in older adults (shown in red square), along with the limitations of psychotherapy (orange square) and safer and more effective non-pharmacological and pharmacological alternatives such as high-intensity physical exercise, electroacupuncture, mindfulness, and α-GPC (green squares). α-GPC: choline alphoscerate; SSRIs: selective serotonin reuptake inhibitors; NRIs: noradrenaline reuptake inhibitors; SNRIs: serotonin and noradrenaline reuptake inhibitors; TCAs: tricyclic antidepressants; MAOIs: monoamine oxidase inhibitors; MDD: major depressive disorder; MCI: mild cognitive impairment; StD: subthreshold depression.

We consider α-GPC a valuable addition to the therapeutic toolkit, especially for older patients (Figure 1). These individuals are often undertreated or managed with medications that fail to provide meaningful benefits. We believe that α-GPC may delay or even prevent the onset of MDD or cognitive impairment for which StD is a known risk factor [43,44,45], offering a new avenue for improving both the quality of life and clinical outcomes in this population.

## 7. Conclusions

In conclusion, α-GPC should be considered in future clinical studies to highlight its therapeutic benefit as monotherapy in StD, particularly in older adults, using the most updated depression scale and assessments as outcome measure, and to verify its potential in preventing or delaying the onset of cognitive symptoms and worsening of depressive symptoms in this population.
